# Optimized design and experimental analysis of the vertical mixing plow for enhancing planosol improvement machine

**DOI:** 10.1038/s41598-025-88083-4

**Published:** 2025-01-27

**Authors:** Jiaxing Du, Jiyao Hou, Yuanyuan Dun, Chuanhua Yang, Xiaohai Li, Baoguo Zhu, Lijun Cai, Guoqiang Dun

**Affiliations:** 1 College of Mechanical Engineering, Jiamusi University, Jiamusi, Heilongjiang China; 2 Jiamusi Branch, Heilongjiang Academy of Agricultural Sciences, Jiamusi, Heilongjiang China; 3 Intelligent Agricultural Machinery Equipment Engineering Laboratory, Harbin Cambridge University, Harbin, Heilongjiang China

**Keywords:** Subsoil compaction, Low nutrient, Root penetration, Tillage depth, Discrete element simulation, Mixing rate, Engineering, Agroecology

## Abstract

Improved soil fertility to sustain crop productivity is important to enhance agroecosystem services. To address the low nutrient content, subsoil compaction, and poor root penetration in Planosol, a new machine was designed to improve these conditions. This machine integrates subsoil mixing and fertilizer application. The paper details the machine’s structure and working principle, focusing on optimizing the vertical mixing plow. Furthermore, discrete element method simulations were conducted, using tillage depth, forward speed, and plow height as factors, with soil mixing rate and tractive resistance serving as the evaluation indicators. Results showed optimal performance at 553 mm tillage depth, 1 m/s speed, and 227 mm plow height, with tractive resistance of 7460.5 N and a soil mixing rate of 72.1%. Field trials and soil improvement tests based on optimized parameters confirmed the accuracy and reliability of the simulation results. Compared to the Shallow plowing and subsoiling area, the subsoil mixing improvement area showed significant improvement, with a yield increase of 16.4–18.9% over two years.

## Introduction

Planosol is one of the primary low-yielding soils in Northeast China^1^, with its low crop productive capacity attributed to both physical and chemical factors. Physically, the Planosol layer is characterized by being dense, hard, and poorly permeable clay-enriched subsoil, forming an obstructive barrier. During crop growth, this layer often presents “hard, compact, and infertile” physical and chemical properties^2,3^, leading to severe surface drought and flooding in the topsoil, and impeding root growth and penetration. Chemically, Planosol is composed of a thin black soil layer with low total nutrient contents, and it becomes increasingly seasonal acidic after cultivation^4^. Therefore, improving and effectively utilizing these soils is crucial for transforming the low-yield characteristics of Planosol areas and boosting crop production with enhanced agroecosystem services.

In recent years, mechanical methods for improving Planosol have been an important issue of agricultural research^5^. Among the most widely adopted techniques in production are deep subsoiling^6^. While these methods have yielded some positive results, they also have significant limitations. Due to the high silt content of Planosol, the soil quickly re-compacts after rain following deep subsoiling^7^, without effectively addressing the fundamental low-yield issues of Planosol. Recognizing the contrasting two-layer structure of Planosol, Zhao et al.^8,9^ proposed a soil improvement method that involves mixing the illuvial horizon with the Planosol layer to reduce subsoil compaction and other related constraints. After years of experimental research, they developed a method of turning over 20 cm of topsoil and mixing it with 30–40 cm of subsoil to create more homogeneous soil conditions. Building on this theory, Araya et al.^10^ and Liu et al.^11^ developed a three-stage subsoil mixing plow. While soil improvement practices have advanced, Zhu et al.^12,13^ successively developed a straw subsoil mixing plow and an subsoil interval mixing plow based on the three-stage subsoil mixing plow. In practical use, the core component, the Vertical Mixing plow, encounters challenges such as high operating resistance, large soil clods, subpar vertical plowing, and inadequate mixing efficiency.

To address these issues and build on the theory of subsoil fertilization and improvement, our research aimed to design a Planosol improvement machine capable of simultaneously mixing subsoil and applying fertilizer. Additionally, the soil mixing principle was refined, and through theoretical analysis combined with discrete element simulation experiments, the structure and operating parameters of the core component, the vertical mixing plow, were optimized. Field validation trials and comparative tests on mechanical soil improvement were conducted.

## Overall structure and working principle

### Overall structure

**Fig. 1:**
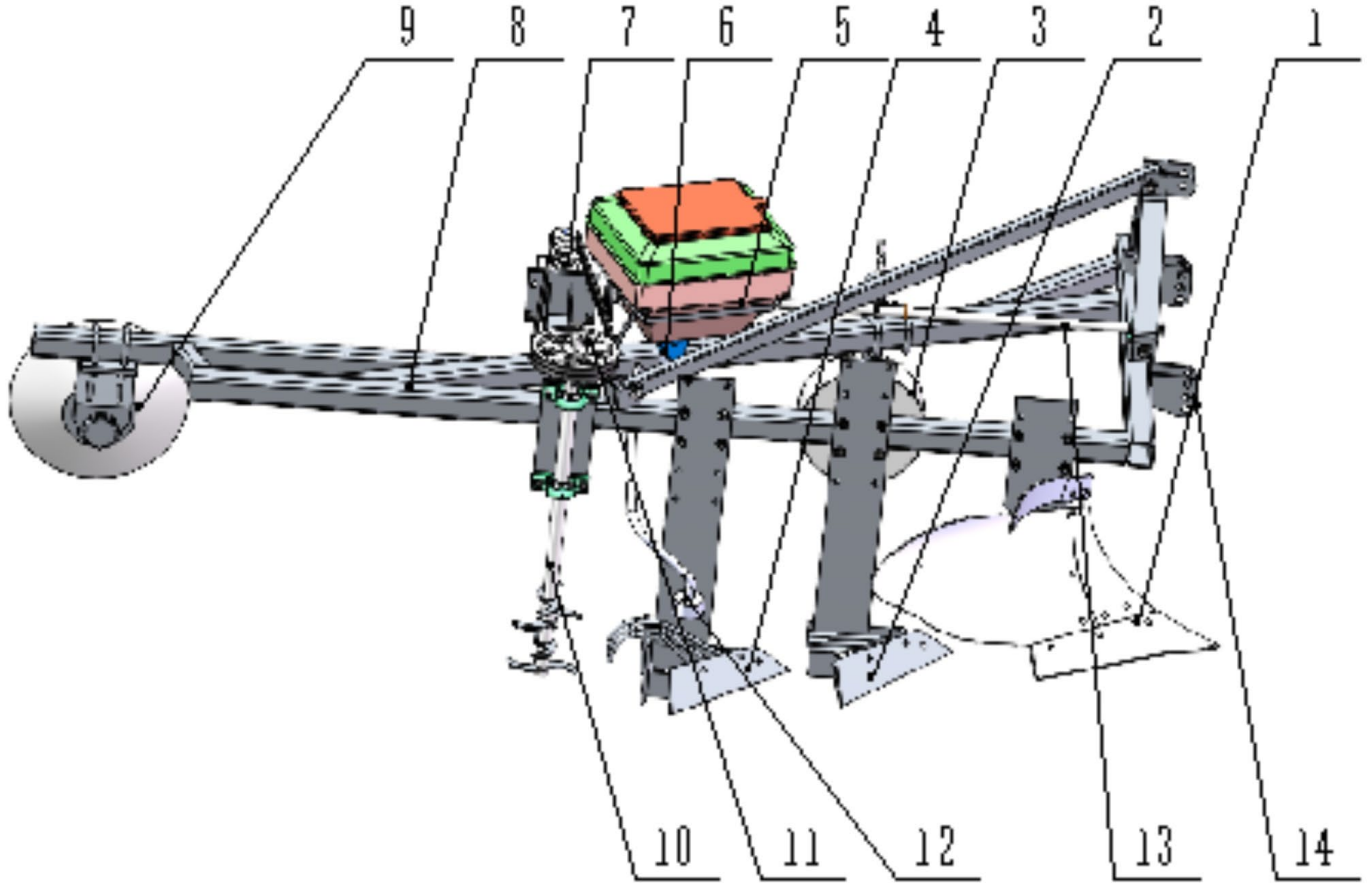
Structure diagram of Planosol improvement machine. (1) tillage layer moldboard plow, (2) Planosol layer crushing plow, (3) ground wheel, (4) vertical mixing plow, (5) fertilizer box, (6) electric fertilizer distributor, (7) gearbox, (8) frame, (9) disc harrow, (10) vertical soil mixer, 11. belt mechanism, 12. fertilizer pipe, 13. transmission shaft, 14. three-point suspension device.

The machine consists of several key components: a tillage layer moldboard plow, a Planosol layer crushing plow, a vertical mixing plow, a fertilization device, a vertical soil mixer, a disc harrow, ground wheels, and a frame (Fig. 1). The components are arranged sequentially on the outer installation beam of the frame, starting from the traction end, as follows: the tillage layer moldboard plow, Planosol layer crushing plow, vertical mixing plow, and vertical soil mixer. The tractor’s output shaft is connected to the machine’s transmission device, which drives the vertical soil mixer for soil mixing operations. This transmission device includes a transmission shaft, a gearbox, and a belt mechanism. The fertilization device consists of a fertilizer box, an electric fertilizer distributor, and fertilizer pipes. A disc harrow is positioned at the rear of the frame to prevent the formation of large soil clods.

## Working principle and main technical parameters

**Fig. 2:**
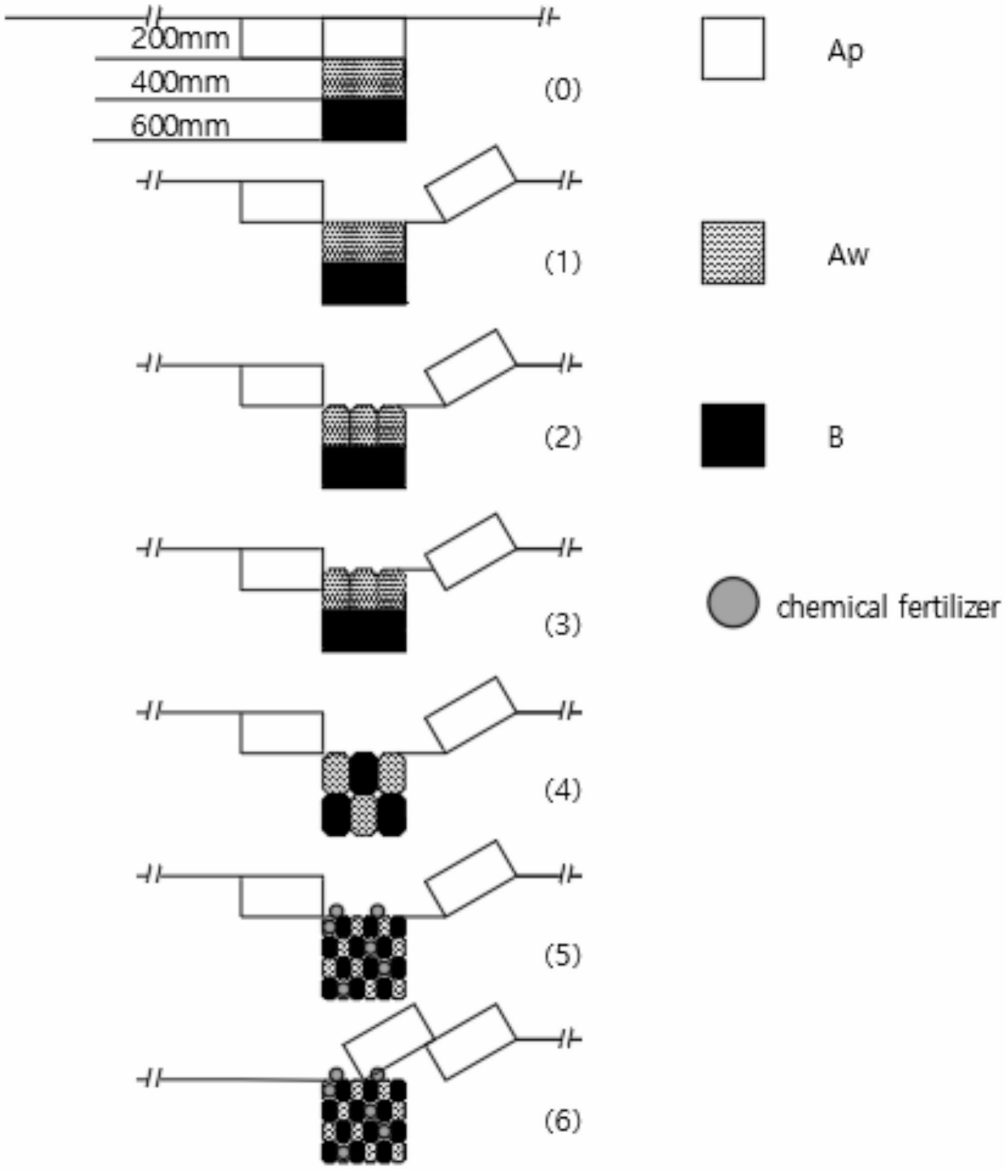
Working principle. Note: Ap: Topsoil; Aw: planosol; B: illuvial horizon. (1)-(6) were working step of plow.

The Planosol improvement machinery is propelled forward by the tractor’s three-point suspension (Fig. 2). The working principles are as follows:

1. The moldboard plow tills the 20 cm topsoil layer (Ap), moving it to one side and creating a 20 cm deep furrow.
2. The Planosol layer crushing plow loosens and crushes the 20 cm Planosol layer (Aw), reducing resistance for subsequent operations.
3. The vertical mixing plow lifts approximately 20 cm of the Planosol layer (Aw) and 10–20 cm of the Illuvial horizon (B), moving it upward along the plow body.
4. The soil clods move downward along the guide bars under the combined forces of gravity and the thrust from the following clod, transitioning from a horizontal to a vertical position before falling back into the furrow. During this process, fertilizer materials are simultaneously applied.
5. The vertical soil mixer further breaks up the vertically oriented soil clods, generating a random mixing effect.
6. During repeated operations, the lifted topsoil fills the previous furrow, leveling it.

The important technical parameters of the Planosol improvement machinery are shown in Table 1.

**Table 1:** Important technical parameters of the Planosol improvement machine.

Parameters	Numerical value
Dimensions (L×W×H) (mm×mm×mm)	3920 × 2500 × 2250
Hook-up method	three-point suspension
Width of tillage layer moldboard plow (mm)	460
Depth of tillage layer moldboard plow (mm)	200 ∼ 300
Width of planosol solum layer crushing plow (mm)	350
Depth of planosol Solum layer crushing plow (mm)	300 ∼ 400
Width of the Vertical Mixing plow (mm)	350
Depth of the Vertical Mixing Plow (mm)	500 ∼ 600
Maximum traction resistance required (kN)	> 35
Vertical soil mixer speed (r/min)	90 ∼ 270
Working speed (m/s)	1 ∼ 2

## Design and analysis of the Vertical Mixing plow

### Design of the Vertical Mixing plow

Given the high adhesion coefficient of the Planosol, the vertical mixing plow is designed with a bar structure to reduce the contact area with the soil, thereby minimizing adhesive force. As the vertical mixing plow moves forward under traction, it lifts approximately 20 cm of the Planosol layer and 10–20 cm of the c The soil is guided upward along the bars to the top of the plow body, where it undergoes fracturing and breaking. Gravity, along with the thrust of subsequent soil clods, then drives the soil downward along the guide bars, transitioning it into a vertical clod state before it falls back into the furrow. The vertical soil mixer follows, breaking and thoroughly mixing the vertically oriented soil clods that have fallen into the furrow.

**Fig. 3:**
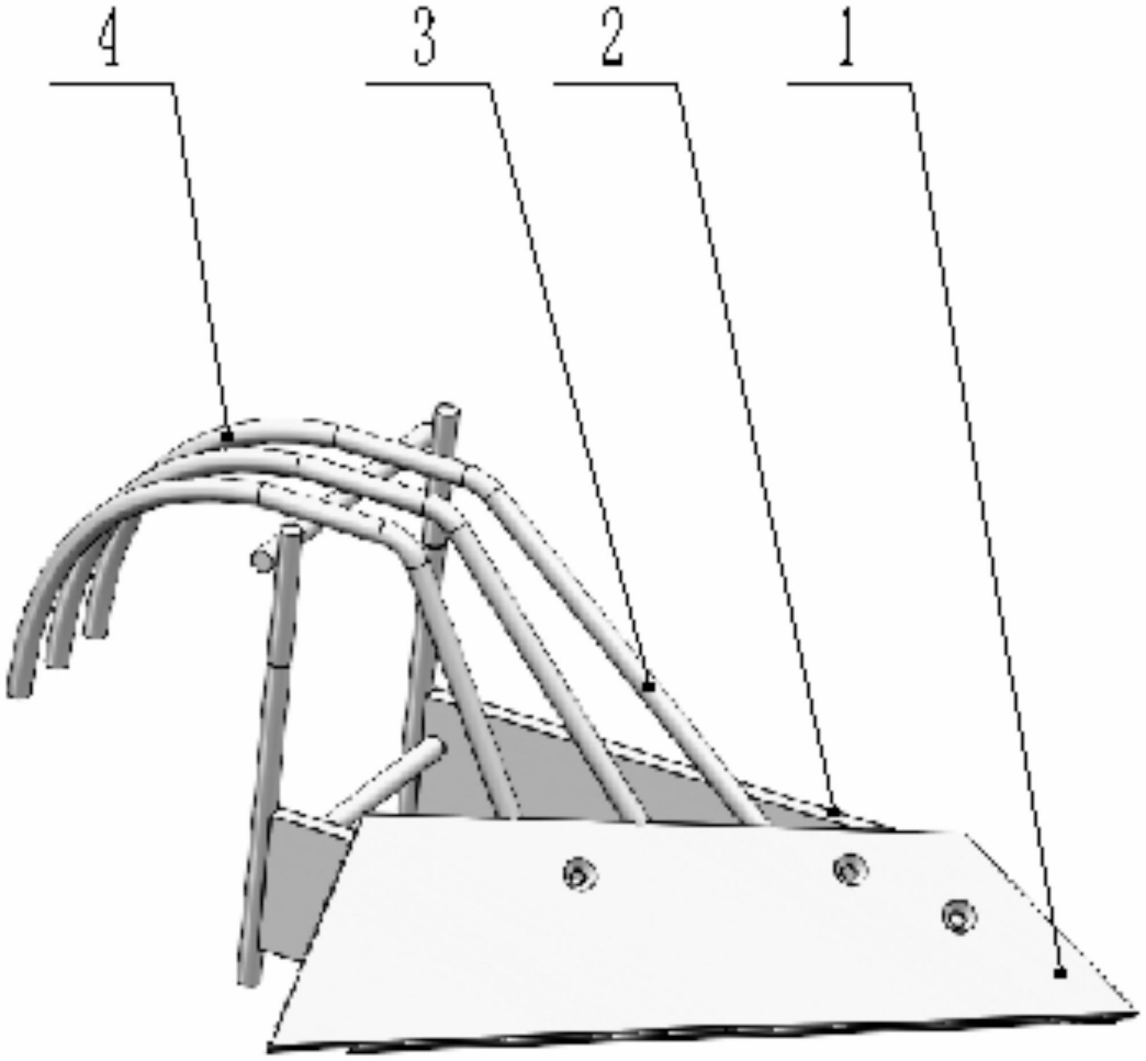
Structure diagram of the vertical mixing plow. (1) plowshare, (2) plow side plate, (3) lifting bars, (4) guiding bars.

The design of the vertical mixing plow includes a plowshare, a plow side plate, lifting bars, and guide bars (Fig. 3). The plowshare is mounted at the front of the main plow body using countersunk screws, allowing for easy replacement when worn. The lifting bars and guiding bars, made of round manganese steel and trapezoidal in shape, are welded to both the plowshare and the plow side plate. This design reduces soil adhesion and enhances the efficiency of the soil mixing process.

## The working principle of the Vertical Mixing plow

**Fig. 4:**
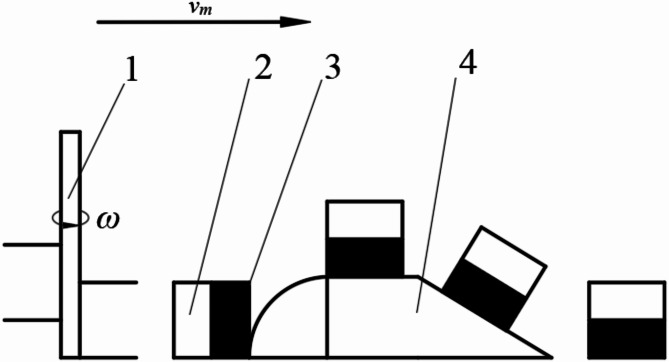
Principle of soil mixing. (1) vertical soil mixer, (2) soil clod of planosol layer, (3) soil clod of the illuvial layer, (4) vertical mixing plow.

As the vertical mixing plow moves forward, the plowshare cuts through the soil clods (Fig. 4). The clods, composed of the Planosol layer and the illuvial horizon, are then pushed along the surface of the plow body by the pressure of the following soil clod, moving toward the top end of the plow body. At the top end, these clods follow a parabolic trajectory along the guide bars, driven by the combined forces of gravity and the thrust from the next clod, falling back into the furrow in a vertical orientation. The vertical soil mixer, positioned behind the vertical mixing plow, then breaks and thoroughly mixes these vertically oriented soil clods.

### Analysis of the Tractive Resistance of the Vertical Mixing Plow

**Fig. 5:**
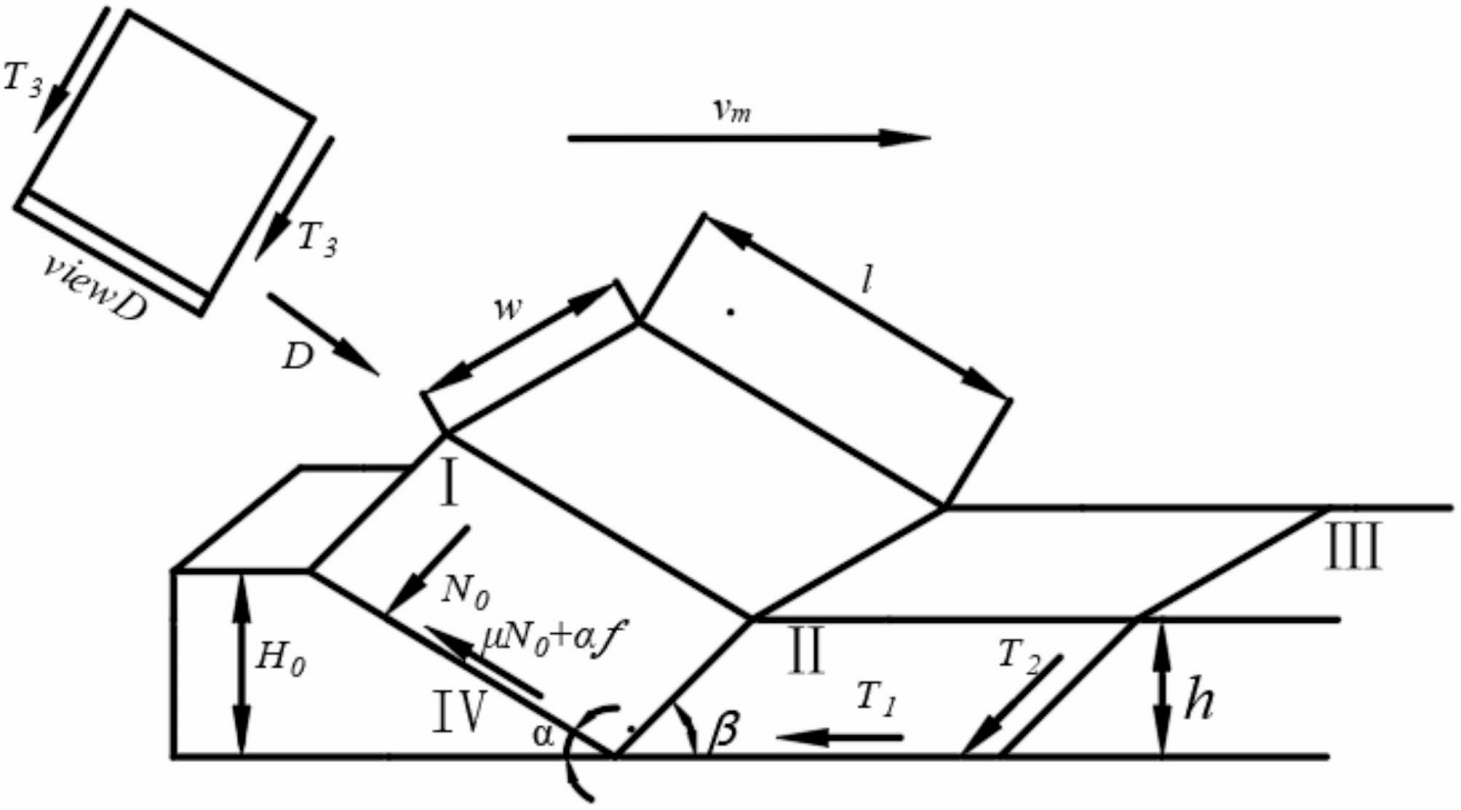
Force analysis diagram of the Vertical Mixing plow. I, II, III soil clods and IV vertical clod mixing plow.

As shown in Fig. 5 the resistance encountered by the Vertical Mixing plow during operation includes the pressure *N*_*0*_ exerted by the clods, the shear resistance $\:{T}_{1}$, $\:{T}_{2}$, $\:{T}_{3}$ of the clods, the frictional resistance $\:u$*N*_*0*_ between the clods and the plow body surface, and the adhesive resistance $\:af$. These components sum up to form the overall resistance vector.

The components of the resistance force on the plow body in the x and y axes, *F*_*x*_ and *F*_*y*_, are shown in Eq. (1)^14^.

$$\:\left\{\begin{array}{c}{F}_{x}={N}_{0}\text{sin}\alpha\:+{N}_{0}u\text{cos}\alpha\:+af\text{cos}\alpha\:+{T}_{1}+{T}_{2}\text{cos}\beta\:\\\:{F}_{y}={N}_{0}\text{cos}\alpha\:+{N}_{0}u\text{sin}\alpha\:+af\text{sin}\alpha\:+{T}_{1}\text{sin}\beta\:\end{array}\:\:\:\:\:\:\:\:\:\:\:\:\:\:\:\:\:\:\:\:\:\:\:\:\:\:\:\:\:\:\:\:\:\:\:\:\:\:\:\:\:\:\:\:\:\:\:\:\:\:\:\:\:\:\:\:\:\:\:\:\:\right.\text{(1)}$$

Where.

*N*_*0*_ is the pressure of the soil on the plow body, N;

$\:\alpha\:$ is the soil entry angle of the plow body, deg;

$\:\beta\:$ is thel angle of crack produced, deg;

$\:u$ is the friction coefficient between the soil and the plow body;

$\:a$ is the adhesion coefficient between the soil and the plow body;

$\:f$ is the contact area between the soil and the plow body, m^2^;

*T*_*1*_ is the shear resistance on the bottom surface of the soil clod, N;

*T*_*2*_ is the shear resistance on the front surface of the soil clod, N;

*T*_*3*_ is the shear resistance on the both side surface of the soil clod, N.

**Fig. 6:**
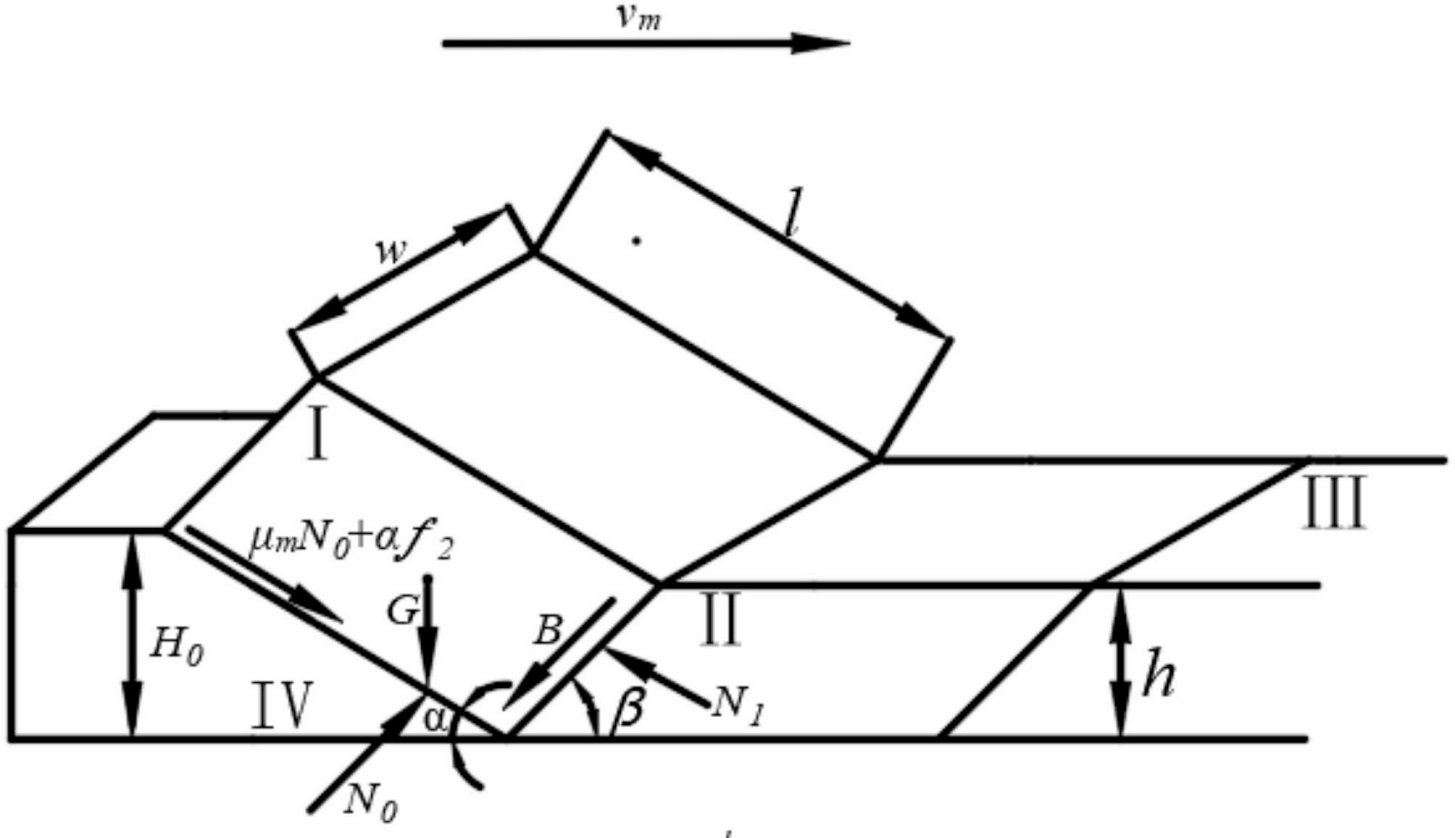
Force analysis diagram of soil clod. I, II, III soil clods and IV vertical clod mixing plow.

As shown in Fig. 6, when the soil clod moves at a constant linear speed on the plow body, the vector sum of the tilled soil block with weight *G*, the supporting force from the plow body *N*_*2*_, the frictional force between the soil clod and the plow body *uN*_*2*_, the adhesive force between the soil clod and the plow body $\:af$, the supporting force from the subsequent soil clod *N*_*1*_, and the inertia force *B* is zero.

The mechanical equilibrium equations of soil clods in the vertical and horizontal directions are shown in Eq. (2).

$$\:\left\{\begin{array}{c}G-{N}_{0}\left(\text{cos}\alpha\:-\mu\:\text{sin}\alpha\:\right)+af\text{sin}\alpha\:+B\text{cos}\beta\:-{N}_{1}\text{cos}\beta\:=0\\\:\left({N}_{2}\text{sin}a+{N}_{2}\mu\:\text{cos}a\right)+af\text{cos}\alpha\:-B\text{cos}\beta\:-{N}_{1}\text{sin}\beta\:=0\end{array}\:\:\:\:\:\:\:\:\:\:\:\:\:\:\:\:\:\:\:\:\:\:\:\:\:\:\:\:\:\:\:\:\:\:\:\:\:\:\:\:\:\:\:\:\:\:\:\:\right.\text{(2)}$$

From Eq. (2), the support force *N*_*2*_ exerted by the plow body on the soil clod and the support force *N*_*1*_ exerted by the subsequent soil clod are shown in Eq. (3):

$$\:\left\{\begin{array}{c}{N}_{2}=\frac{G\text{sin}\beta\:-af\text{cos} (\alpha\:+\beta\:) +B}{\text{sin}(\alpha\:+\beta\:)+\mu\:\text{cos}(\alpha\:+\beta\:)}\\\:{N}_{1}=\frac{{N}_{2}\left(\text{sin}\alpha\:+\beta\:\right)+af\text{cos}\alpha\:-B\text{cos}\beta\:}{\text{sin}\beta\:}\end{array}\:\:\:\:\:\:\:\:\:\:\:\:\:\:\:\:\:\:\:\:\:\:\:\:\:\:\:\:\:\:\:\:\:\:\:\:\:\:\:\:\:\:\:\:\:\:\:\:\:\:\:\:\:\:\:\:\:\:\:\:\:\:\:\:\:\:\:\:\:\:\:\:\:\:\:\:\:\:\:\:\:\:\:\right.\text{(}\text{3}\text{)}$$

The expressions for the weight of the soil clod *G*, inertial force *B*, shear resistance *T*_*1*_ on the bottom surface of the soil clod, shear resistance *T*_*2*_ at the front of the soil clod, shear forces *T*_*3*_ on both sides of the soil clod, and the contact area $\:f$between the soil clod and the plow body, as referenced in Eq. (1) to (3), are given in Eq. (4)^15^.

$$\:\left\{\begin{array}{c}G=\frac{h{H}_{0}w{\rho\:}_{b}g}{{sin}a}\\\:B={\rho\:}_{b}wh{\left({v}_{m}{cos}\beta\:\right)}^{2}{\:T}_{1}=\pi\:wh{\sigma\:}_{t}{\xi\:}_{1}\\\:{T}_{2}=\frac{\pi\:w{H}_{0}{\sigma\:}_{t}{\xi\:}_{2}}{{sin}\alpha\:}\\\:{T}_{3}=\frac{\pi\:h{H}_{0}{\sigma\:}_{t}{\xi\:}_{3}}{{sin}a}\\\:f=\frac{{H}_{0}w}{{sin}\alpha\:}\end{array}\text{\:\:\:\:\:\:\:\:\:\:\:\:\:\:\:\:\:\:\:\:\:\:\:\:\:\:\:\:\:\:\:\:\:\:\:\:\:\:\:\:\:\:\:\:\:\:\:\:\:\:\:\:\:\:\:\:\:\:\:\:\:\:\:\:\:\:\:\:\:\:\:\:\:\:\:\:\:\:\:\:\:\:\:\:\:\:\:\:\:\:\:}\text{(4)}\right.$$

Where.

*N*_*1*_ is the supporting force from the subsequent soil clod, N;

*N*_*2*_ is supporting force from the plow body, N;

*B* is the inertia force, N;

*G* is the weight of soil block produced, N.

$\:h$ is the tillage depth, mm;

*w* is width of plow blade, mm;

$\:{\rho\:}_{b}$ is the bulk density of the soil, kg/m^3^;

$\:\text{g}$ is the acceleration due to gravity, m/s^2^.

$\:{\sigma\:}_{t}$ is the shear strength of the soil;

$\:\:{\xi\:}_{1,}\:{\xi\:}_{2}\:{,\xi\:}_{3}$is the correction coefficients;

*v*_*m*_ is the forward speed, m/s;

*H*_*0*_ is the height of the plow body, mm.

**Fig. 7:**
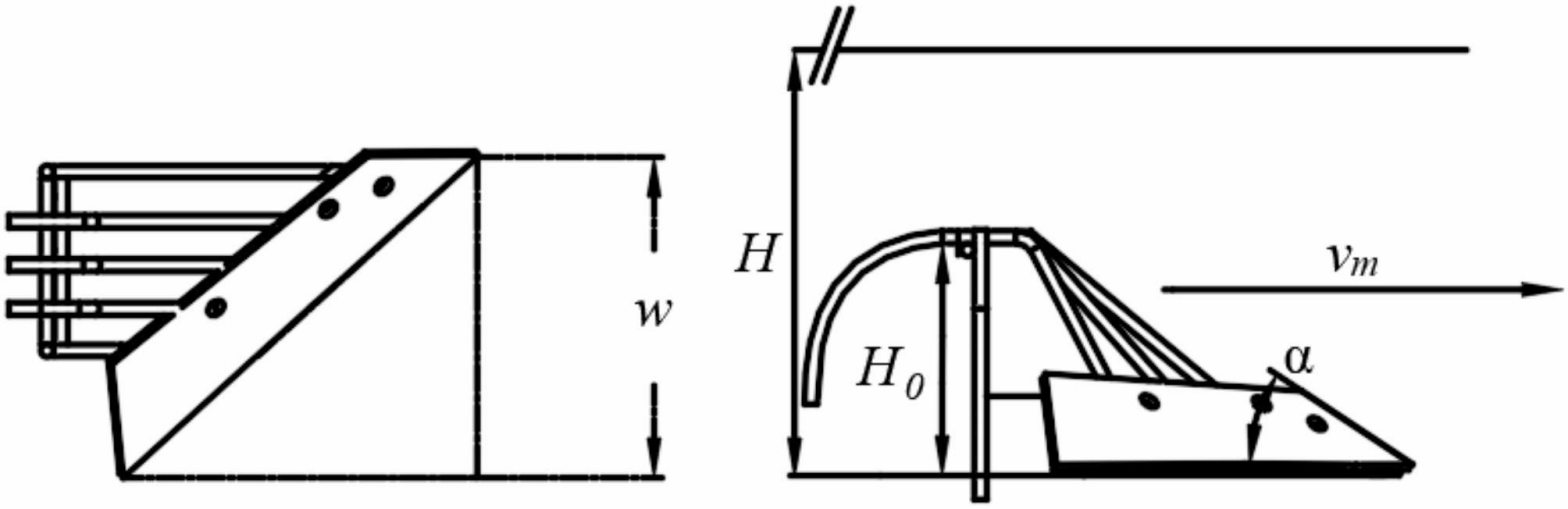
Operational parameters diagram of vertical mixing plow.

The primary factors affecting the resistance experienced by the vertical mixing plow include the soil entry angle of the plow body (α), tillage depth (*H*), width of the plow blade (*w*), forward speed (*v*_*m*_), and plow body height (*H*_*0*_) (Fig. 7). Based on previous design structures, the width of the plow blade is set to 350 mm, and the soil entry angle of the plow body is set to 30°. The tillage depth (*H*), plow body height (*H*_*0*_), and forward speed (*v*_*m*_) require further analysis to be determined.

## Analysis of Soil Mixing Effect

**Fig. 8:**
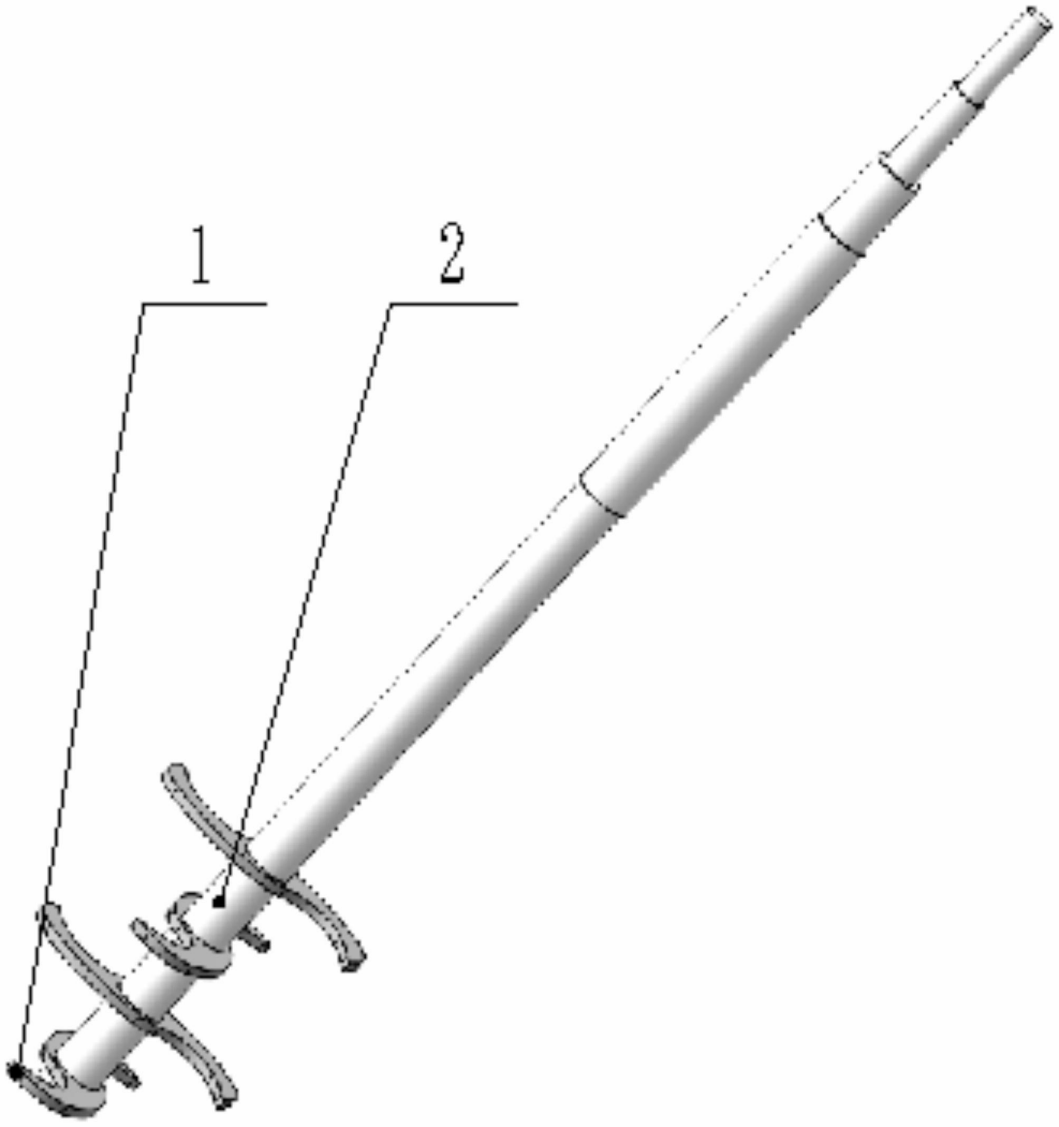
Structural diagram of a vertical soil mixer. (1) vertical mixing blade, (2) rotating shaft.

The vertical soil mixer consists of multiple vertical mixing blades mounted on a rotating shaft (Fig. 8). The vertical mixing blade is right-handed with a rotation diameter of 300 mm. Within a working height of 325 cm, it is equipped with four blade tools arranged perpendicularly and staggered. Power is supplied by the tractor’s output shaft, which transmits power to the rotating shaft through a universal joint, transmission shaft, gearbox, and pulley, thereby powering the vertical soil mixer.

**Fig. 9:**
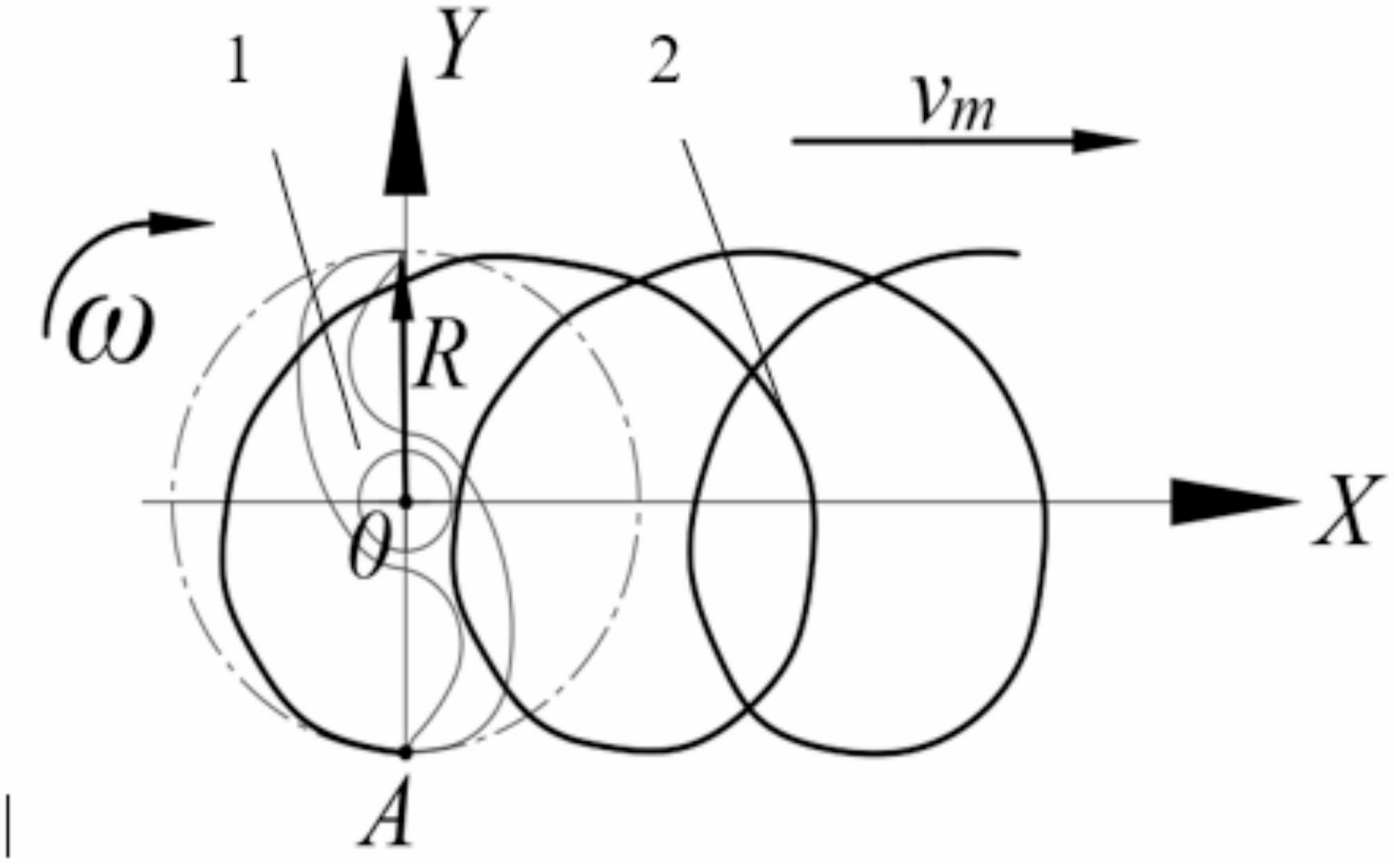
Movement trajectory diagram of vertical mixing knife. 1.Vertical mixing blade.

Based on the different speed ratios *λ* between the circumferential speed of the blade tip and the forward speed of the machine, where *λ* = *Rω*/*v*_*m*_, the motion trajectory of the working parts is determined. The initial rotation center of the vertical blade is taken as the origin, with the forward direction as the *X*-axis, and the direction parallel to the ground and perpendicular to the *X*-axis as the *Y*-axis. A coordinate system is established as shown in Fig. 9.

2.Motion trajectory.

The motion equation of point A is shown in Eq. (5):

$$\:\left\{\begin{array}{c}x=R\text{cos}\omega\:t+{v}_{0}t\\\:y=R\text{sin}\omega\:t\end{array}\right.\:\:\:\:\:\:\:\:\:\:\:\:\:\:\:\:\:\:\:\:\:\:\:\:\:\:\:\:\:\:\:\:\:\:\:\:\:\:\:\:\:\:\:\:\:\:\:\:\:\:\:\:\:\:\:\:\:\:\:\:\:\:\:\:\:\:\:\:\:\:\:\:\:\:\:\:\:\:\:\:\:\:\:\:\:\:\:\:\:\:\:\:\:\:\:\:\:\:\:\:\:\:\:\:\:\:\:\:\:\:\:\:\:\:\:\:\:\:\:\:\:\:\:\:\:\:\text{(}\text{5}\text{)}$$

The partial velocity of point A in the x-axis and y-axis direction is:

$$\:\left\{\begin{array}{c}{v}_{x}={v}_{m}-60R\omega\:\text{sin}\omega\:t\\\:{v}_{y}=R\omega\:\text{cos}\omega\:t\end{array}\right.\text{\:\:\:\:\:\:\:\:\:\:\:\:\:\:\:\:\:\:\:\:\:\:\:\:\:\:\:\:\:\:\:\:\:\:\:\:\:\:\:\:\:\:\:\:\:\:\:\:\:\:\:\:\:\:\:\:\:\:\:\:\:\:\:\:\:\:\:\:\:\:\:\:\:\:\:\:\:\:\:\:\:\:\:\:\:\:\:\:\:\:\:\:\:\:\:\:\:\:\:\:\:\:\:\:\:\:\:\:\:\:\:\:\:\:\:\:\:\:}\text{(6)}$$

Where.

*R* is the distance from point A to the center of rotation, mm;

$\:\omega\:$ is the angular speed of the vertical stirring knife, rad/s;

*v*_*m*_ is the forward speed, m/s;

*t* is the operation time, s.

The main factors affecting the operational effectiveness of the vertical soil mixer include the forward speed, the rotational speed, and the number and distribution of the blades. Based on virtual simulations and preliminary tests, when the working width is 350 mm, the diameter of the vertical soil mixer is 300 mm, and the rotational speed is 330 r/min, it ensures that the fallen soil clods are fully cut, mixed, and spread at the bottom of the trench. When the rotational speed is 330 r/min, the forward speed of the machine should be chosen between 1 and 2 m/s.

**Fig. 10:**
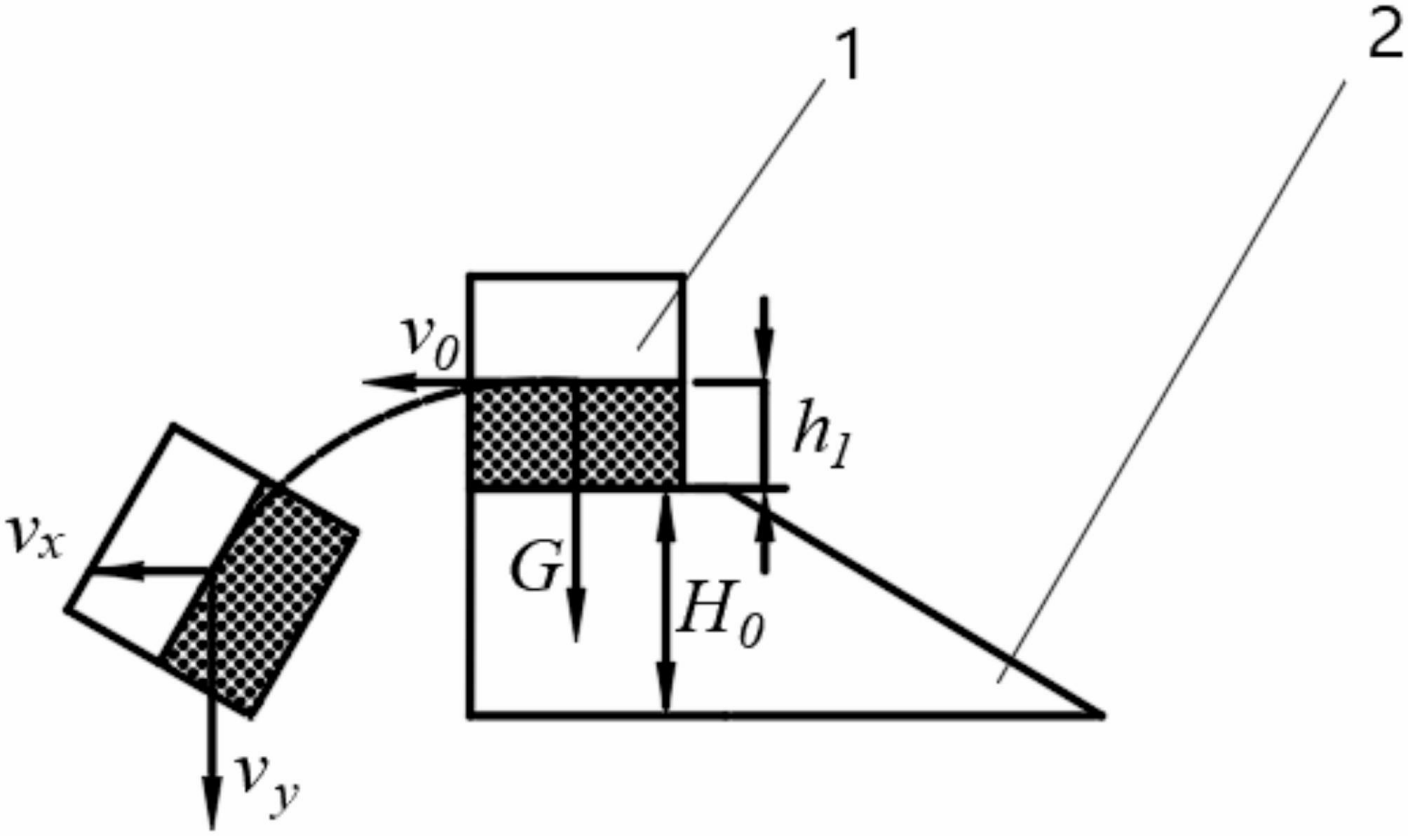
Analysis of soil clod movement. (1) soil clod, (2) vertical mixing plow.

As shown in Fig. 10, the soil clods move backward relative to the plow. Upon leaving the plow, they acquire an initial horizontal velocity $\:{v}_{0}$. Under the influence of resistance, the soil clods undergo decelerated linear motion in the horizontal direction, while simultaneously undergoing free fall motion in the vertical direction due to gravity.

As the soil clod moves along a parabolic trajectory, its velocity at any point is the vector sum of $\:{v}_{x}$ and $\:{v}_{y}$. Over time, $\:{v}_{x}$ gradually decreases while $\:{v}_{y}$increases. The closer the angle between the direction of the soil clod’s velocity and the vertical direction approaches 0°, the closer the soil clod is to an upright state. When the height of the vertical soil-mixing plow increases within a certain range, the time it takes for the soil clod to reach the bottom of the furrow also increases, making the soil clod more upright when it reaches the bottom. This results in better mixing during the operation of the vertical soil-mixing plow; however, the resistance encountered by the plow body also increases. Referring to the design of previously studied soil-mixing plows^11,12^, the height *H*_*0*_ of the vertical soil-mixing plow is selected within the range of 200 to 300 mm.

As the plowing depth increases, more illuvial horizon soil will rise along the plow body surface together with the Planosol layer, turning into upright soil clods and thereby improving the mixing rate. However, the increase in plowing depth also leads to an increase in tractive resistance. Therefore, the plowing depth *H* of the vertical mixing plow should be chosen between 500 mm and 600 mm.

## Simulation experiment

### Establishment of discrete element method simulation model

To accommodate the size of the machine, a soil bin with dimensions of 4000 mm (length) × 1500 mm (width) × 600 mm (height) was established in EDEM.

The equipment designed in this study is intended for operation under the Planosol conditions in Northeast China. To ensure the accuracy of the experiment, spherical particles with varying parameters for the topsoil layer, Planosol layer, and illuvial horizon were used to simulate the respective soil layers, with each layer having a thickness of approximately 200 mm. In the simulation, spherical particles with a radius of 10 mm were employed as the soil particle model. To better replicate the hardness and compaction of Planosol, the particle contact model was set as Hertz-Mindlin with bonding, with a bonding radius of 10.5 mm. Each layer contained 170,000 particles, resulting in a total of 510,000 particles. The discrete element simulation parameters were determined using a combination of experimental measurements and literature references^16–18^, with the specific parameters detailed in Table 2.

**Table 2:** Simulation parameters.

Parameters			value
Material parameters	Topsoil layer	Poisson’s ratio	0.42
		Density/(kg/m^3^)	1304
		Modulus of Shear/(Pa)	1.38 × 10^6^
	Planosol layer	Poisson’s ratio	0.4
		Density/(kg/m^3^)	1987
		Modulus of Shear/(Pa)	1.38 × 10^6^
	Illuvial horizon	Poisson’s ratio	0.29
		Density/(kg/m^3^)	1902
		Modulus of Shear/(Pa)	1.23 × 10^6^
	steel	Poisson’s ratio	0.35
		Density/(kg/m^3^)	7830
		Modulus of Shear/(Pa)	7.27 × 10^10^
Contact parameters	Soil-soil	coefficient of Restitution	0.5
		coefficient of Static friction	0.8
		coefficient of Rolling friction	0.23
	Soil-steel	coefficient of Restitution	0.6
		coefficient of Static friction	0.6
		coefficient of Rolling friction	0.05
Contact model parameters	Topsoil layer	Normal stiffness per unit area /(N/m^3^)	2.4 × 10^7^
		Shear stiffness per unit area /(N/m^3^)	1.6 × 10^7^
		Normal critical stress /(Pa)	2 × 10^6^
		Critical shear stress /(Pa)	3.8 × 10^5^
	Planosol layer	Normal stiffness per unit area /(N/m^3^)	3.8 × 10^7^
		Shear stiffness per unit area /(N/m^3^)	2.4 × 10^7^
		Normal critical stress /(Pa)	2.3 × 10^6^
		Critical shear stress /(Pa)	6.8 × 10^5^
	Illuvial horizon	Normal stiffness per unit area /(N/m^3^)	3.4 × 10^7^
		Shear stiffness per unit area /(N/m^3^)	1.2 × 10^7^
		Normal critical stress /(Pa)	1.4 × 10^6^
		Critical shear stress /(Pa)	5.7 × 10^5^

**Fig. 11:**
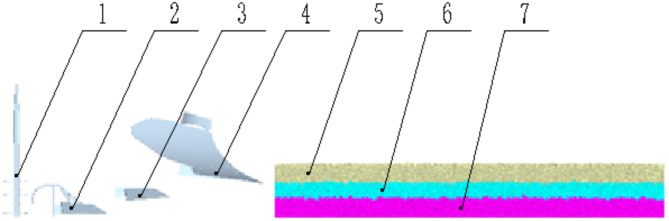
Simulation Model. (1) vertical soil mixer, (2) vertical mixing plow, (3) crushing plow, (4) moldboard plow, (5) topsoil layer soil particles, (6) planosol layer soil particles, (7) illuvial horizon particles.

After constructing the soil bin model, the three-dimensional drawing software SolidWorks was used to create solid models of the Planosol improvement machine’s soil-contact components: the tillage layer moldboard plow, the Planosol layer crushing plow, the Vertical Mixing plow, and the vertical soil mixer. These models were imported into the EDEM software. The forward speed, tillage depth, vertical soil mixer rotation speed, and simulation time were set to obtain the simulation model (Fig. 11).

### Experimental design and evaluation criteria

#### Experimental design

Based on the above analysis, simulation experiments were designed using the Box-Behnken central composite design principle. The three factors—forward speed, tillage depth of the Vertical Mixing plow, and height of the Vertical Mixing plow—were taken as independent variables. The soil particle mixing rate of the Planosol layer and the Illuvial horizon, and the traction resistance of the Vertical Mixing plow, were used as experimental indicators. The coding of each experimental factor is shown in Table 3.

**Table 3:** Coding of test factors.

Code	Factor		
	Forward speed X_1_ (m/s)	Tillage depth X_2_ (mm)	Plow height X_3_ (mm)
−1	1	500	200
0	1.5	550	250
−1	2	600	300

### Evaluation criteria

(1) The traction resistance experienced by the vertical soil-mixing plow.

**Fig. 12:**
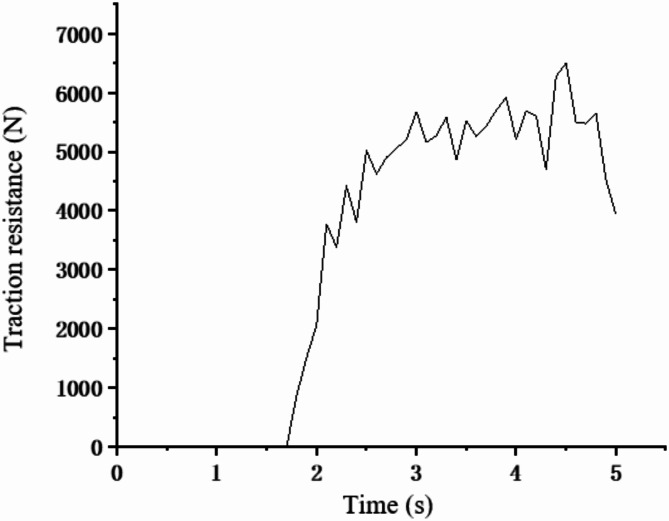
Tractive Resistance of vertical soil-mixing plow.

As shown in Fig. 12, the period from 2.5 to 4.5 s after the operation stabilizes is selected as the statistical object. Using the resistance measurement feature in EDEM 2018 post-processing, the traction resistance curve experienced by the vertical soil-mixing plow during its operation is generated. The average traction resistance within the operating region is then calculated and used as the experimental response value, denoted as *Y1*.

(2) Soil mixing rate.

**Fig. 13:**
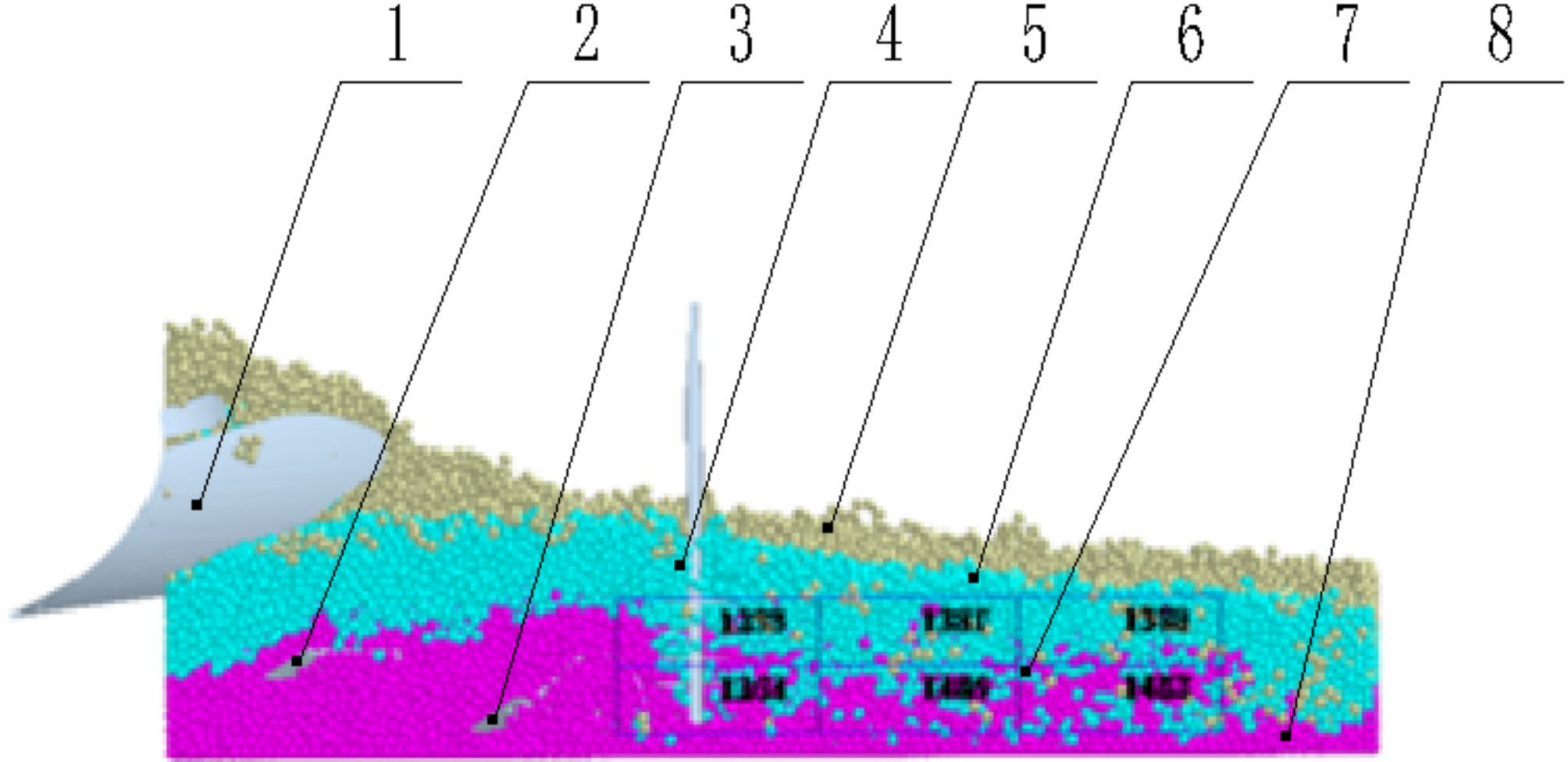
Virtual simulation process of EDEM. (1) topsoil layer moldboard plow, (2) planosol layer crushing plow, (3) vertical mixing plow, (4) vertical soil mixer, (5) topsoil layer soil particles, (6) planosol layer soil particles, (7) monitoring area, (8) illuvial horizon particles.

As shown in Fig. 13, the simulation process involves selecting the 2000 mm region after the operation has stabilized for statistical analysis. Using the Geometry Bin function in the Setup Selection of EDEM post-processing, a monitoring area of 2000 mm in length, 350 mm in width, and 400 mm in height is defined. This monitoring area is then divided into 18 equal sub-monitoring zones, each approximately 667 mm in length, 117 mm in width, and 200 mm in height. The number of original Planosol layer particles, (*f*_*1*_ to *f*_*18*_) and the total number of particles(*t*_*1*_ to *t*_*18*_) are then recorded for each sub-monitoring zone.

Referring to the theories of powder technology^19,20^, the mixing rate M is calculated using the following formula:

7 $$\:M=1-{\left(\delta\:/{\delta\:}_{0}\right)}^{2}\:\:\:\:\:\:\:\:\:\:\:\:\:\:\:\:\:\:\:\:\:\:\:\:\:\:\:\:\:\:\:\:\:\:\:\:\:\:\:\:\:\:\:\:\:\:\:\:\:\:\:\:\:\:\:\:\:\:\:\:\:\:\:\:\:\:\:\:\:\:\:\:\:\:\:\:\:\:\:\:\:\:\:\:\:\:\:\:\:\:\:\:\:\:\:\:\:\:\:\:\:\:\:\:\:\:\:\:\:\:\:\:\:\:\:\:\:\:\:\:\:\:\:\:\:\:\:\:\:\:\:$$

Where

$\:{\delta\:}^{2}$ is the variance of the ratio of Planosol layer soil particles to the total number of particles in the monitoring zone after operation compared to the ideal ratio.

$\:{\delta\:}_{0}^{2}\:\:$is the variance of the ratio of Planosol layer soil particles to the total number of particles in the monitoring zone before operation compared to the ideal ratio.

8 $$\:{\delta\:}^{2}=\sum\:_{i=1}^{\text{18}}(f\text{i}/\text{t}\text{i}-1/2)/\text{18}\:\:\:\:\:\:\:\:\:\:\:\:\:\:\:\:\:\:\:\:\:\:\:\:\:\:\:\:\:\:\:\:\:\:\:\:\:\:\:\:\:\:\:\:\:\:\:\:\:\:\:\:\:\:\:\:\:\:\:\:\:\:\:\:\:\:\:\:\:\:\:\:\:\:\:\:\:\:\:\:\:\:\:\:\:\:\:\:\:\:\:\:\:\:\:\:\:\:\:\:\:\:\:\:\:\:\:\:\:\:\:\:\:\:$$

9 $$\:{\delta\:}_{0}^{2}=\text{9}\left[{\left(1-\frac{1}{2}\right)}^{2}+{\left(0-\frac{1}{2}\right)}^{2}\right]/\text{18}=\text{0.25}\:\:\:\:\:\:\:\:\:\:\:\:\:\:\:\:\:\:\:\:\:\:\:\:\:\:\:\:\:\:\:\:\:\:\:\:\:\:\:\:\:\:\:\:\:\:\:\:\:\:\:\:\:\:\:\:\:\:\:\:\:\:\:\:\:\:\:\:\:\:\:\:\:\:\:\:\:\:\:\:\:\:\:\:\:\:\:\:$$

The calculated soil mixing rate M is taken as the response value *Y*_*2*_ for the experiment.

### Simulation Test results and analysis

#### Simulation Test results

A total of 17 sets of response surface analysis experiments were conducted, with *X*_*1*_, *X*_*2*_, and *X*_*3*_ representing the coded values of the factors. The response surface design experiment scheme and results are shown in Table 4.

**Table 4:** Test plan and experimental results.

Test number	Factor			Y_1_ Tractive resistance/*N*	Y_2_ Mixing rate/%
	X_1_	X_2_	X_3_		
1	−1	−1	0	7971.6	69.3
2	1	−1	0	9088.6	58.6
3	−1	1	0	8614.9	67.4
4	1	1	0	9799.4	68.5
5	−1	0	−1	6536.3	66.6
6	1	0	−1	6941.4	52.4
7	−1	0	1	10274.4	74.3
8	1	0	1	14958.3	63.4
9	0	−1	−1	6981	52.7
10	0	1	−1	8944.3	62.5
11	0	−1	1	10,231	67.1
12	0	1	1	13,882	72.3
13	0	0	0	8799.5	67.6
14	0	0	0	8673.6	68.4
15	0	0	0	8414.5	68.5
16	0	0	0	7847.3	71.5
17	0	0	0	9454.3	69.5

Using Design-Expert 10.0.1 software, the fitting analysis of the factors was conducted^21,22^. The regression models for the tractive resistance (*Y*_*1*_) generated by the vertical mixing plow and the mixing rate (*Y*_*2*_ ) were subjected to analysis of variance. The results of the analysis are presented in Tables 5 and 6.

The P-value of the quadratic regression model for tractive resistance is 0.004, indicating that the regression model is highly significant (Table 5). The lack-of-fit term P-value is greater than 0.05, and the R² value is 0.92, indicating that the lack-of-fit is not significant and that the regression model demonstrates a high degree of fit.The influence of experimental factors on tractive resistance, from greatest to least, is the height of the vertical mixing plow, the tillage depth of the vertical mixing plow, and the forward speed. The significant terms are, *X*_*1*_, *X*_*2*_, *X*_*3*_, and *X*_*3*_^*2*^. The other terms are not significant and were removed. The equation was re-evaluated to establish the final regression model as follows:

**Table 5:** Variance analysis of tractive resistance.

Source	Sum of squares	df	Mean square	F	*P*
Model	7.34 × 10^7^	9	8.16 × 10^6^	9.21	0.0040**
*X*_*1*_	6.83 × 10^6^	1	6.83 × 10^6^	7.71	0.0274*
*X*_*2*_	6.07 × 10^6^	1	6.07 × 10^6^	6.85	0.0345*
*X*_*3*_	4.97 × 10^7^	1	4.97 × 10^7^	56.13	< 0.0001**
*X*_*1*_ * × *_*2*_	1139.06	1	1139.06	0.0013	0.9724
*X*_*1*_ * × *_*3*_	4.58 × 10^6^	1	4.58 × 10^6^	5.17	0.0572
*X*_*2*_ * × *_*3*_	7.12 × 10^5^	1	7.12 × 10^5^	0.804	0.3997
*X*_*1*_^*2*^	10778.33	1	10778.33	0.0122	0.9153
*X*_*2*_^*2*^	3.33 × 10^5^	1	3.33 × 10^5^	0.3764	0.5589
*X*_*3*_^*2*^	5.01 × 10^6^	1	5.01 × 10^6^	5.65	0.0491*
Residual	6.20 × 10^6^	7	8.86 × 10^5^		
Lack of Fit	4.83 × 10^6^	3	1.61 × 10^6^	4.71	0.0844
Pure Error	1.40 × 10^6^	4	3.42 × 10^5^		
Cor Total	7.96 × 10^7^	16			

Note: ** indicates highly significant (*P* *≤* 0.01),* indicates significant (0.01 *≤* *P* *≤* 0.05).

10 $$\:{Y}_{1}=\text{8740.4}\text{1}+\text{923}\text{.8}\text{1}{X}_{1}+\text{871.0}\text{5}{X}_{2}+\text{2}\text{492}\text{.84}{X}_{3}+1103.18{X}_{3}^{2}\:\:\:\:\:\:\:\:\:\:\:\:\:\:\:\:\:\:\:\:\:\:\:\:\:\:\:\:\:\:\:\:\:\:\:\:\:$$

From Table 6, it can be seen that the P-value of the quadratic regression model for the mixing rate is 0.0041, indicating that the regression model is highly significant. The lack-of-fit p-value is 0.0596, slightly above the 0.05 threshold. However, with an R² value of 0.92, the model demonstrates a good fit. In EDEM simulations, the precision of simulation parameters is often difficult to guarantee. Considering the complexity of the soil model and the variability of real-world conditions, minor fitting errors may occur. This indicates that the regression model is still acceptable. The influence of experimental factors on the mixing rate, from greatest to least, is the height of the Vertical Mixing plow, forward speed, and the tillage depth of the Vertical Mixing plow. The significant terms are *X*_*1*_, *X*_*2*_, *X*_*3*_, and *X*_*3*_^*2*^. The other terms are not significant and were removed. The equation was re-evaluated to establish the final regression model as follows:

**Table 6:** Variance analysis of mixing rate.

Source	Sum of squares	df	Mean square	F	*P*
Model	572.87	9	63.65	9.1	0.0041**
*X*_*1*_	150.51	1	150.51	21.52	0.0024**
*X*_*2*_	66.13	1	66.13	9.45	0.0179*
*X*_*3*_	230.05	1	230.05	32.89	0.0007**
*X*_*1*_ * × *_*2*_	34.81	1	34.81	4.98	0.0609
*X*_*1*_ * × *_*3*_	2.72	1	2.72	0.39	0.5525
*X*_*2*_ * × *_*3*_	5.29	1	5.29	0.76	0.4133
*X*_*1*_^*2*^	7.25	1	7.25	1.04	0.3424
*X*_*2*_^*2*^	14.22	1	14.22	2.03	0.197
*X*_*3*_^*2*^	54.95	1	54.95	7.86	0.0264*
Residual	48.96	7	6.99		
Lack of fit	39.94	3	13.31	5.9	0.0596
Pure Error	9.02	4			
Cor total	621.82	16			

Note: ** indicates highly significant (*P* *≤* 0.01),* indicates significant (0.01 *≤* *P* *≤* 0.05).

11 $$\:{Y}_{2}=\text{67.70}-\text{4.34}{X}_{1}+\text{2.88}{X}_{2}+\text{5.36}{X}_{3}-\text{3.79}{X}_{3}^{2}\:\:\:\:\:\:\:\:\:\:\:\:\:\:\:\:\:\:\:\:\:\:\:\:\:\:\:\:\:\:\:\:\:\:\:\:\:\:\:\:\:\:\:\:\:\:\:\:\:\:\:\:\:\:\:\:\:\:\:\:\:\:\:\:\:\:\:$$

### Response surface analysis

**Fig. 14:**
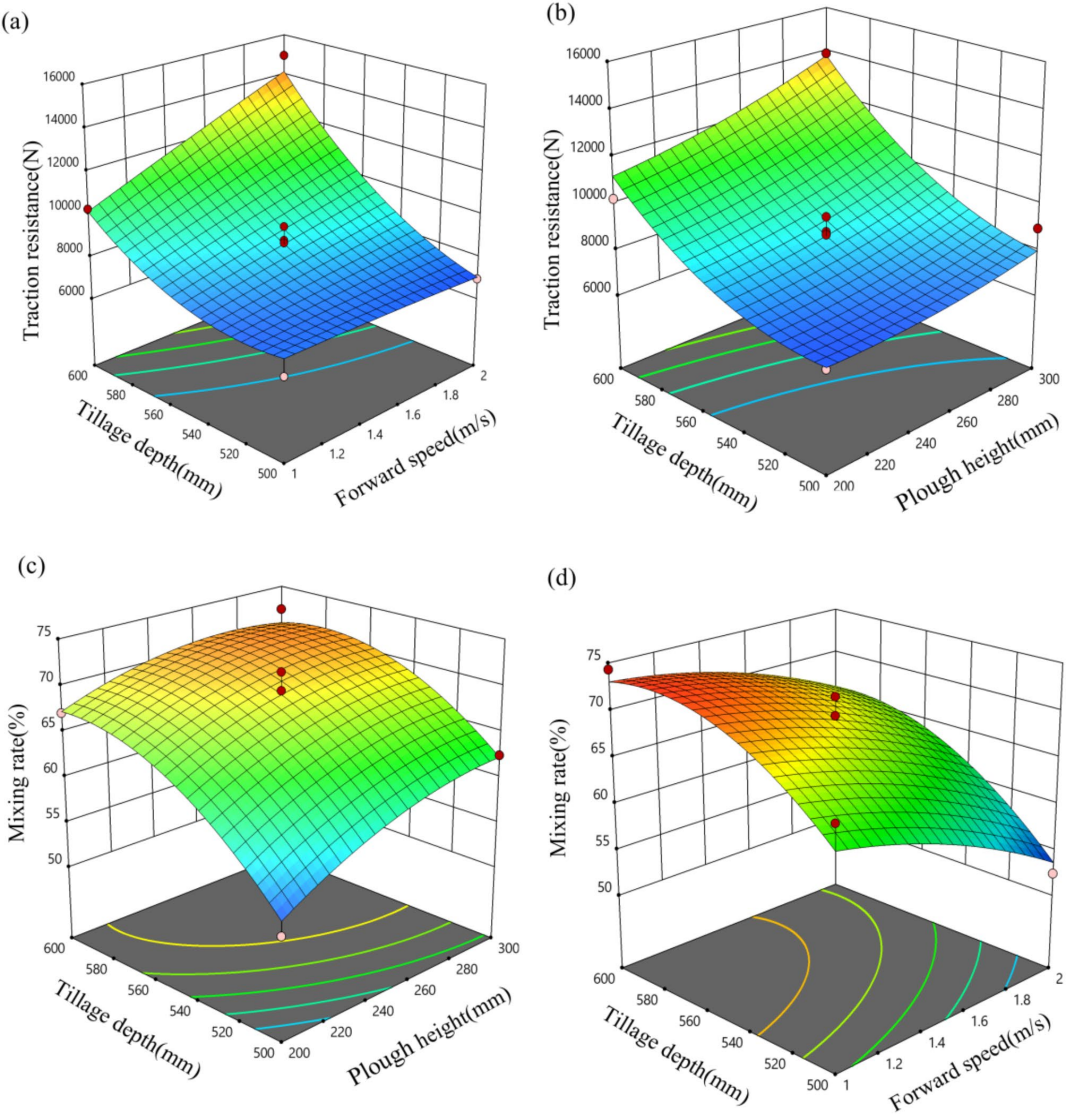
Response surface under different factor interactions.

Using Design-Expert 10.0.1 software to analyze the data, response surfaces depicting the interaction effects of forward speed, tillage depth, and plow height of the Vertical Mixing plow on tractive resistance and soil mixing rate were generated (Fig. 14).

Figure 14(a) shows the response surface of the tractive resistance of the Vertical Mixing plow with respect to the tillage depth and forward speed. When the forward speed is constant, the tractive resistance of the Vertical Mixing plow significantly increases as the tillage depth increases within the range of 500 mm to 600 mm. Similarly, when the tillage depth is constant, the tractive resistance increases continuously as the forward speed increases within the range of 1 to 2 m/s.

Figure 14(b) illustrates the interactive response surface of the tractive resistance of the Vertical Mixing plow with respect to the tillage depth and the height of the Vertical Mixing plow. When the height of the Vertical Mixing plow is constant, the tractive resistance significantly increases as the tillage depth increases within the range of 500 to 600 mm. Similarly, when the tillage depth is constant, the tractive resistance continuously increases as the height of the Vertical Mixing plow increases within the range of 200 to 300 mm.

Figure 14(c) shows the interactive response surface of the soil mixing rate with respect to the tillage depth and the height of the Vertical Mixing plow. When the height of the Vertical Mixing plow is constant, the soil mixing rate initially increases and then stabilizes as the tillage depth increases within the range of 500 to 600 mm. Similarly, when the tillage depth is constant, the soil mixing rate also initially increases and then stabilizes as the height of the Vertical Mixing plow increases within the range of 200 to 300 mm.

Figure 14(d) shows the interaction response surface plot of the effects of the tillage depth and forward speed of the vertical mixing plow on soil mixing rate. When the forward speed is constant, the soil mixing rate initially increases and then stabilizes as the tillage depth of the Vertical Mixing plow increases within the range of 500 to 600 mm. Conversely, when the tillage depth of the Vertical Mixing plow is constant, the soil mixing rate continuously decreases as the forward speed of the equipment increases within the range of 1 to 2 s.

Using Design-Expert 10.0.1 software, the optimal solution for the Vertical Mixing plow, which minimizes traction resistance and maximizes mixing rate, was determined as follows: the tillage depth of the Vertical Mixing plow of 553 mm, a forward speed of 1 m/s, and at the height of the Vertical Mixing plow of 227 mm. Under these conditions, the theoretical traction resistance of the Vertical Mixing plow was calculated to be 7460.5 N, with a mixing rate of 72.1%. For practical implementation, the Vertical Mixing plow was designed with the tillage depth of the Vertical Mixing plow of 550 mm, at the height of the Vertical Mixing plow of 230 mm, and a forward speed of 1 m/s.

### Field validation

#### Experimental equipment and conditions

The experimental equipment and instruments include a trial model of the Planosol improvement machine, a standard shallow plowing and subsoiling plow, Dongfanghong 1904 and 1404 tractors, towing ropes, towing rings, measuring tapes, a laptop, a DIK-5521 penetration-type soil hardness tester, a ring knife assembly, an NTJL-7 (100kN) tension sensor, and the accompanying TG ONE force measurement data acquisition software.

**Fig. 15:**
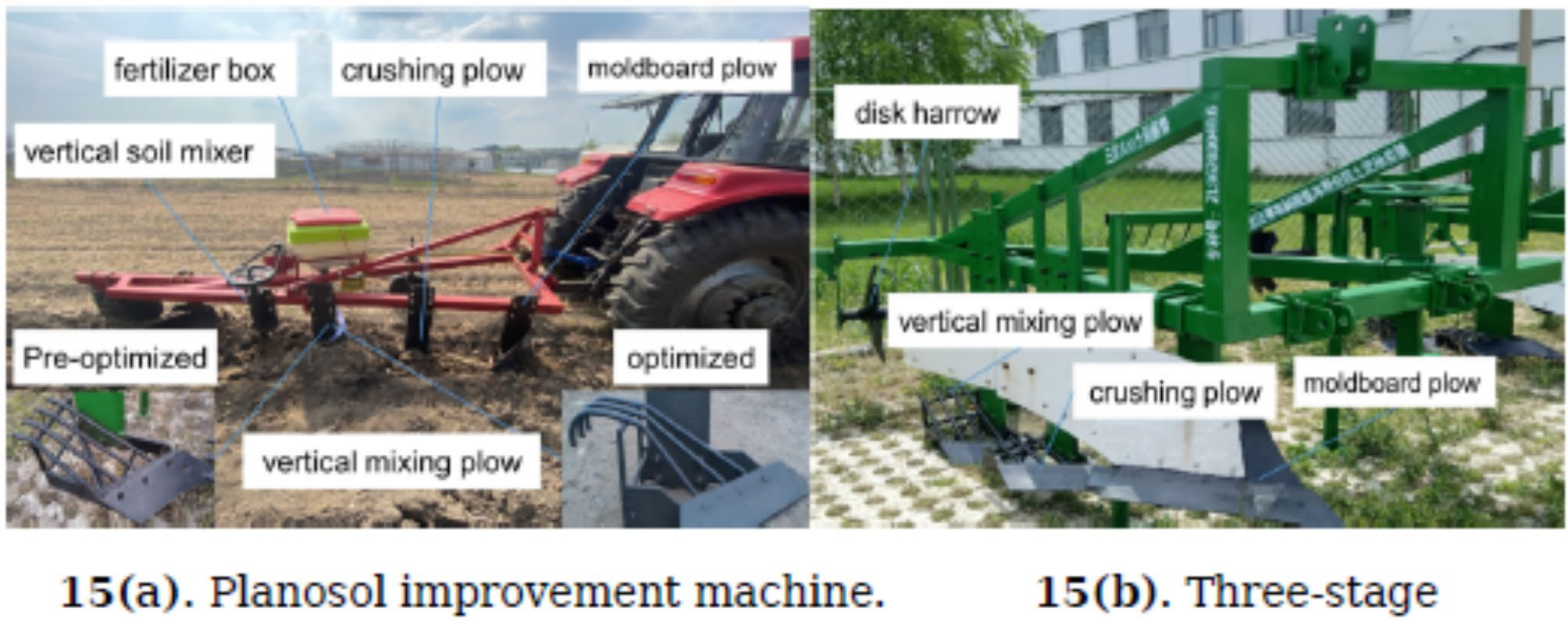
Operation performance verification test.

In October 2021, at the dryland experimental station of the 854 State Farm in Heilongjiang Province, performance verification and comparative trials were conducted on the Planosol improvement machine (Fig. 15). Figure 15(a) shows the three-stage subsoil mixing plow, while Fig. 15(b) presents the optimized planosol improvement machine. Compared to the traditional three-stage subsoil mixing plow, the improved design features added guiding bars at the rear of the subsoil mixing plowshare and further optimized structural dimensions, including an increased plow height. The newly added vertical soil mixer performs three key functions: further soil crushing, mixing, and leveling of the trench bottom, effectively preventing the formation of large soil clods. Additionally, a layered fertilization device was incorporated, enabling simultaneous fertilization of the planosol layer and the topsoil layer. The experiment adopted continuous positioning measurements, with the tested soil type being planosol.

The comparative soil improvement trial included two treatments: a conventional shallow plowing and subsoiling area (CK) using a shallow plowing and subsoiling plow and a Subsoil mixing area (MX) using the Planosol improvement machine. Each treatment covered an area of 0.2 hectares. The test field’s previous crop was corn, continuously planted for three years. Soybeans were planted in the experimental area in 2022 and 2023 without further soil tillage operations.

### Experimental method

#### Tractive resistance

**Fig. 16:**
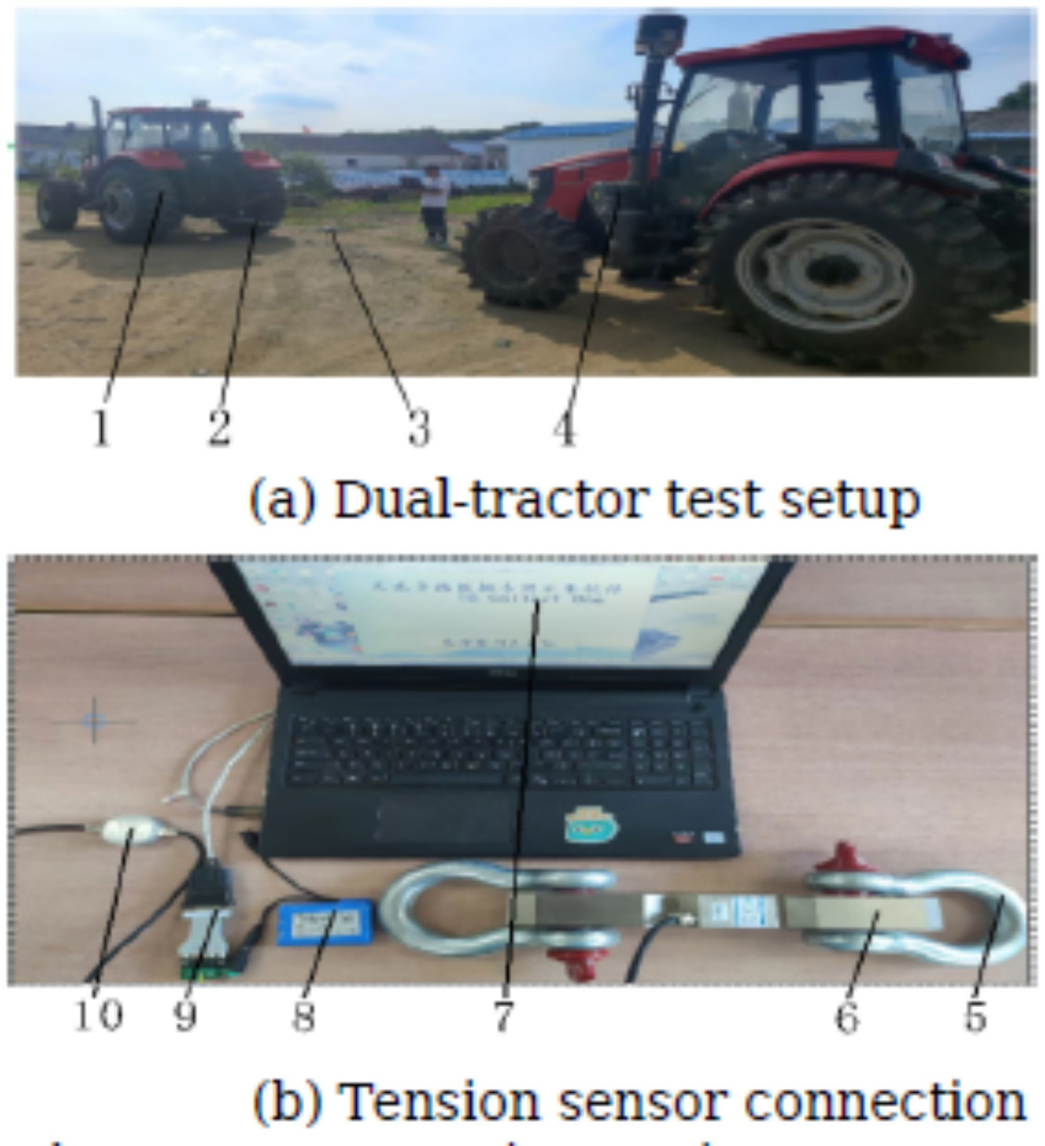
Tractive resistance test system. (1) Dongfanghong 1904 Tractor, (2) Towing Cable, (3) Traction Resistance Testing System, (4) Dongfanghong 1404 Tractor, (5) Towing Ring, (6) NTJL-7 Load Cell, (7) TG ONE Data Acquisition Software, (8) Digital Module Sensor Power Supply, 9.RS485 Converter Cable, 10, Digital Module Sensor.

Tractive resistance test system, as shown in Fig. 16. The Dongfanghong 1904 model tractor operates in first gear at the front, while the Dongfanghong 1404 model tractor, attached to the different soil improvement machinery, remains in neutral. The NTJL-7 (100kN) tension sensor is connected between the two tractors using towing rings and towing ropes. The traction resistance is measured both with and without the vertical mixing plow attached, and the difference between the two measurements represents the experimental traction resistance, as illustrated in Fig. 16(a).

The NTJL-7 (100kN) tension sensor is connected to a laptop via an RS485-to-USB data cable. Real-time traction resistance data from the digital module sensor is recorded using the TG ONE data acquisition software installed on the laptop. The traction resistance values are measured over a stable 100-meter stretch within the test soil improvement area, and the average value is calculated.as shown in Fig. 16(b).

### Soil mixing rate

In the mechanical soil improvement test area, soil profiles were excavated every 30 m to observe the soil mixing effect, resulting in three profiles in total. After natural air-drying, the original Planosol layer soil appeared grayish-white, forming a clear contrast with the black deposition layer. The horizontal soil profile images were processed using the ImageJ software to convert them to grayscale, and the black area (S1) and white area(S2) within the original Planosol layer were quantified.

The soil mixing rate *M* between the planosol layer soil and the illuvial horizon soil is calculated as follows:

12 $$\:M=\frac{\text{m}\text{i}\text{n}({S}_{1},{S}_{2})}{\text{m}\text{a}\text{x}({S}_{1},{S}_{2})}\:\times\:100\%\:\:\:\:\:\:\:\:\:\:\:\:\:\:\:\:\:\:\:\:\:\:\:\:\:\:\:\:\:\:\:\:\:\:\:\:\:\:\:\:\:\:\:\:\:\:\:\:\:\:\:\:\:\:\:\:\:\:\:\:\:\:\:\:\:\:\:\:\:\:\:\:\:\:\:\:\:\:\:\:\:\:\:\:\:\:\:\:\:\:\:\:\:\:\:\:\:\:\:\:\:\:\:\:\:\:\:\:\:\:\:\:\:\:\:\:\:\:$$

Where.

min(*S*_*1*_,*S*_*2*_) is the smaller value between *S*_*1*_and *S*_*2*_.

max(*S*_*1*_,*S*_*2*_) is the larger value between *S*_*1*_ and *S*_*2*_.

Using this method, the average mixing rate of the three soil profiles was calculated and used as the evaluation indicator for soil mixing rate.

#### Soil Physical properties

Soil bulk density and moisture content were measured using the ring knife sampling method and the drying method. Soil profiles with dimensions of 100 × 100 × 100 cm were manually excavated, with three profiles in total. Ring knife sampling was conducted at three layers: 0–20 cm, 20–40 cm, and 40–60 cm. Each layer was sampled three times at each profile, and the average value was calculated. Soil hardness was determined using a DIK-5521 penetration-type soil hardness tester equipped with a cone head with a base area of 2 cm². A 10-point positioning method was used to measure the soil hardness of the 0–60 cm soil layer in the test area, and the average value was recorded.

### Yield measurement

Yield was measured through a full-area harvest.

#### Experimental results and analysis

##### Traction resistance and soil mixing rate

The test results are shown in Table 7. The results indicate that the soil mixing performance of the Planosol improvement machine is significantly better than that of the three-stage subsoil mixing plow. After tillage, no large soil clods were observed, and the soil mixing rate increased by 9.4%. Although the improved vertical mixing plow features additional guiding bars, their impact on traction resistance is minimal and can be considered negligible.

**Table 7:** Mechanical operation performance results.

Machinery Type	Working Width /cm	Working Depth /cm	Working Efficiency /(hm^2^/h)	Vertical Mixing Plow Traction Resistance/*N*	Soil Mixing Rate(%)
Three-stage subsoil mixing plow	50	53 ~ 60	0.2 ~ 0.4	7150	60.5
Planosol improvement machine	46	50 ~ 60	0.2 ~ 0.4	7747.7	69.9

#### Soil physical properties

In September 2023, soil physical properties were measured, and the results are shown in Table 8. One of the primary causes of low productivity in Planosols is the excessive hardness of the Planosol layer, which hinders root penetration. After soil improvement operations, the hardness of the mixed 20–40 cm Planosol layer decreased from 20 to 30 kg/cm² to 10–15 kg/cm² compared to the control, while the bulk density was reduced by 0.2 g/cm³. Moreover, the soil moisture content in the 20–40 cm layer was significantly higher than that in the control, with an increase of 4.6%. These findings indicate that Subsoil mixing significantly improved soil permeability and water retention, creating a more favorable environment for crop growth.

**Table 8:** Soil physical properties before and after mechanical soil improvement.

Treatments	Soil Layer Depth (cm)	Soil Bulk Density (kg/cm³)	Soil Moisture Content (%)	Average Hardness (kg/cm²)
Shallow plowing and subsoiling (CK)	0 ~ 20(Ap)	1.25	19.9	3 ~ 10
	20 ~ 40(Aw)	1.48	17.6	20 ~ 30
	40 ~ 60(B)	1.40	19.6	15 ~ 25
Subsoil mixing (MX)	0 ~ 20(Ap)	1.17	20.4	3 ~ 6
	20 ~ 40(Aw)	1.28	22.2	10 ~ 15
	40 ~ 60(B)	1.35	20.1	15 ~ 20

### Yield improvement

As shown in Table 9, compared to the control, the Planosol improvement machine increased yield by 18.9% in the first year and 16.4% in the second year compared to the conventional shallow plowing and subsoiling plow. This indicates that the Planosol improvement machine demonstrates a more significant and long-lasting effect in improving soil and enhancing crop growth conditions, primarily due to the complete disruption of the restrictive Planosol layer.

**Table 9:** Experiment on yield improvement.

Years	Treatments	Yield/(kg/hm^2^)	Increasing Yield/%
2022	Shallow plowing and subsoiling(CK)	2060.2	——
	Subsoil mixing(MX)	2450.5	18.9
2023	Shallow plowing and subsoiling(CK)	2345.5	——
	Subsoil mixing(MX)	2731.4	16.4

## Discussion

1. According to the local agricultural machinery department’s assessment, the cost of conventional surface soil tillage is approximately 30 yuan per standard acre. The annual cost of subsoiling is equivalent to 2 standard acres, about 60 yuan. The one-time cost of the Planosol improvement machine is 100 yuan, equivalent to 3.3 standard acres, with an annual cost of about 20 yuan over 5 years for ongoing soil improvement. The annual cost of subsoiling is 60 yuan. Under the assumption that other maintenance and operational costs are the same, mechanical soil improvement saves 40 yuan per year, resulting in a total savings of 200 yuan per acre over 5 years.
2. This study only focused on a specific type of Planosol. There are significant differences in soil properties and the timing of soil improvement operations under different climate conditions and in different regions, which may affect the improvement results. Future research should include a wider range of Planosol types to more comprehensively assess the applicability and effectiveness of soil improvement techniques.
3. The current prototype can only perform single-line soil improvement with a working width of 46 cm, resulting in low operational efficiency. While it can complete the soil improvement task in one pass, it is not yet capable of large-scale, rapid soil improvement. Therefore, future design improvements should focus on enhancing operational efficiency and expanding the working width to provide technical support for Planosol mechanized soil improvement.

## Conclusions

1. To enhance the effectiveness of mechanized Planosol soil improvement, a Planosol improvement machine was designed based on the theory of subsoil fertilization and improvement. The core component, the Vertical Mixing Plow, was optimized. The study identified the main factors affecting the traction resistance and soil mixing effectiveness of the Vertical Mixing Plow during operation.
2. The soil mixing and improvement process was simulated using EDEM software. The experiment factors included the tillage depth of vertical mixing plow, forward speed, and height of the vertical mixing plow, while the experiment indicators were the traction resistance and soil mixing rate. A regression mathematical model was established between the factors and indicators. When the machine’s forward speed was 1 m/s, the vertical mixing plow height was 230 mm, and the tillage depth of the Vertical Mixing plow was 550 mm, the theoretical traction resistance was 7460.5 N, and the soil mixing rate was 72.1%. Field validation experiments showed that the actual field traction resistance was 7747.7 N, and the mixing rate was 69.9%, with errors of 4% and 3% compared to the theoretical values, respectively, confirming the accuracy of the simulation results. tillage depth of the Vertical Mixing plow.
3. The mechanical soil improvement control tests show that the Planosol improvement machine effectively breaks the Planosol layer, significantly increasing soil moisture content, while reducing bulk density and hardness. The changes in soil properties are particularly noticeable in the 20–40 cm soil layer. Over the soil improvement benefit period (2 years), the yield increase ranged from 16.4 to 18.9%. The machine demonstrates good soil mixing performance, outperforming traditional Planosol improvement machinery.

## Data Availability

The data presented in this study are available on request from the corresponding author.
